# Emission Reduction Benefits and Economic Benefits of China's Pilot Policy on Carbon Emission Trading System

**DOI:** 10.1155/2022/5280900

**Published:** 2022-04-15

**Authors:** Zhijia Wang, Lijuan Liang, Dong Cheng, Hujun Li, Yongheng Zhang

**Affiliations:** ^1^Research Center for Energy Economic, Henan Polytechnic University, Jiaozuo 454000, China; ^2^School of Finance and Economics, Henan Polytechnic University, Jiaozuo 454000, China; ^3^School of Business, Guilin Tourism University, Guilin 541006, China; ^4^School of Civil Engineering, Henan Polytechnic University, Jiaozuo 454000, China

## Abstract

As a market means to control nongreen behaviors of firms, the most expected incentive effect of the carbon emission trading system (CETS) is to achieve the dual economic and environmental effects. As a typical developing country, whether China's CETS has a positive incentive effect is significant to controlling greenhouse gas. Based on the quasinatural experiment of China's pilot policy on CETS in 2013, this study investigates its emission reduction and economic benefits using the difference-in-difference (DID) method. Then, the realization mechanism of CETS's incentive benefits is reversely studied with the idea that goals generate behavior. The results show that the following: (a) China's CETS has produced positive incentive effects of promoting both economic and emission reduction benefits. Furthermore, the results are still valid after using the instrumental variable to overcome the endogenous problem, placebo tests to eliminate sampling bias, and a series of robustness tests. (b) Further analysis shows that firms can choose to improve technology innovation and energy efficiency to get the positive incentive effects of CETS. (c) The incentive effects of CETS also have regional heterogeneity. The emission reduction and economic benefits are greater in provinces with deficient resource endowments and strict environmental law enforcement.

## 1. Introduction

Since the 21st century, climate warming has become a major obstacle to global sustainable development. Therefore, the United Nations proposed the carbon emission trading system (CETS) to regulate greenhouse gas emissions and alleviate the contradiction between ecological protection and economic development. According to “*Kyoto Protocol*,” this market-oriented environmental regulation (ER) was first applied in developed countries. Since European Union (EU) launched the world's largest CETS in 2005, CETS has been widely recognized for its role in curbing greenhouse gas emissions and promoting economic development [1–3]. However, some scholars argued that CETS could fail to bring out its best incentive benefits due to the imperfect trading and management systems [4–6]. Therefore, current research still focuses on whether CETS can produce positive incentive effects, especially how to produce and get the best incentive effects. Apart from developed countries, developing countries like China and India also need to reduce greenhouse gas emissions. After four decades of rapid economic growth, China became the world's second-largest economy in 2010. However, the rapid economic growth has also caused serious environmental problems. According to Carbon Brief, China emitted 10. 1 bn tons of CO_2_ in 2018, the highest in the world, and 1.2 bn tons more than the sum of the US (5.4 bn tons) and the EU (3.5 bn tons). It can be seen that China plays a vital role in the world's economic and environmental problems. Studying the incentive benefits of China's CETS can provide substantial evidence for carbon reduction.

The research ideas of this article are as follows: Firstly, this study verifies whether China's CETS have positive incentive effects under the guidance of the new behavior theory. Secondly, the realization path is explored to obtain positive incentive effects. Finally, the heterogeneity of incentive effects is analyzed to provide more accurate information for the effective implementation of CETS. The specific methods are as follows: Firstly, China's pilot policy on CETS in 2013 is used as a quasinatural experiment to analyze its emission reduction and economic benefits using the DID method. CO_2_ emissions and total factor productivity (TFP) are used to represent the emission reduction benefits and economic benefits, respectively. Dynamic benefits analysis, instrumental variables, counterfactual test, placebo test, and other methods are used to test the robustness of the constructed econometric model. That is, to test whether the emission reduction benefits and economic benefits of the pilot policy on CETS are still significant after overcoming endogeneity, policy implementation time, and selection bias of pilot areas. Secondly, the mediation model is used to examine whether CETS's positive incentive effects can be achieved through technological innovation and energy efficiency. Finally, grouping regression is adopted to investigate CETS's incentive effects heterogeneity.

The marginal contributions of this study are as follows: (a) This study applies behavior theory to the research of ER. The role of China's market regulation is investigated from the perspective of the incentive effect. The realization path of incentive effect is discussed with the idea that goals generate behavior. This study provides a new vision for the formulation and implementation of ER. (b) This study includes emission reduction and economic benefits in a unified research framework. In the context of China, a more comprehensive evaluation of CETS's policy benefits is made. The ability of CETS to balance the environment and economy in developing regions is tested, which provides evidence for the decrease of greenhouse gas. (c) The empirical analysis based on the quasinatural experiment and DID avoid endogenous problems such as missing variables to a certain extent. However, it still could not eliminate the interference of regional characteristics that changed over time. Therefore, this paper further tests the robustness of the empirical results through instrumental variables, counterfactual test, and placebo test. It provides scientific evidence for studying emission reduction benefits and economic benefits of CETS.

The article is organized as follows.  Section 2 presents the literature review.  Section 3 analyzes the institutional background and theory.  Section 4 discusses the results of the empirical research.  Section 5 presents the results of the mechanism analysis.  Section 6 presents highlights conclusions and the scope of future work.

## 2. Literature Review

### 2.1. Research on the Benefits of ER

The relationship between ER and economics is controversial in academia. The neoclassical economic theory supports the view that ER internalizes pollution as cost and transfers resources from production to environmental protection [7, 8]. Therefore, the firms' production efficiency may regress in the short term [9]. However, economists represented by Porter raised objections. Porter and van der Linder [10] proposed the Porter hypothesis, which elaborates that strict and appropriate ER can generate higher productivity through innovation. The hypothesis has been widely discussed. Lanoie et al. [11] and Peuckert [12] believed that the positive effects of ER through environmental technology could offset the short-term cost and ultimately benefit production efficiency. Testa et al. [13] found that more flexible ER significantly increased R&D investment and eventually improved firms' production efficiency. Using the steel industry data, Liu et al. [14] proved that economic incentives significantly boosted firms' profitability. Using panel data of 17 manufacturing industries, Rubashkina et al. [15] found that productivity increased only in industries under ER. With the increasing international status of China, scholars pay more attention to the environmental and economic benefits of ER in the context of China. Li et al. [16] found that the promotion effects of China's ER on production efficiency is only significant in the eastern provinces. Li and Chen [17] studied the promotion effects of China's air pollution prevention and control law on TFP by DID model. Wang and Liu [18] proved an inverted N relationship between China's ER and TFP.

### 2.2. Research on CETS

Research on CETS focus on policy effects. They generally use developed countries as data sources. Scholars believed that CETS has positive policy effects. Anderson et al. [19] confirmed the emission reduction effects of EU's CETS in the manufacturing industry and firms participating in CETS are more likely to implement green technology innovation. However, some scholars believe that the policy effects of CETS are limited. Hoffmann [20] compared the emission reduction effects of the 2008 economic crisis and the CETS, and the results showed the former were far greater than the latter. Borghesi et al. [21] conducted an empirical study based on Italy's manufacturing industry and found that overly loose allocation of carbon quota limited the policy effects of the EU CETS. While China's CETS has a short running time, its emission reduction and economic benefits are unclear. Findings of the emission reduction benefits are inconsistent. Li and Zhang [22] proved that China's pilot policy on CETS significantly suppressed carbon emissions using industrial data. However, affected by the heterogeneity of objects, carbon quota, carbon price, and regional policies, the emission reduction benefits of CETS are not always significant [23, 24]. Findings of the economic benefits are also controversial. The empirical results from Liao et al. [25] suggested that the CETS generated green economic benefits by stimulating green innovation. Wang and Wang [26] found that China's CETS did not significantly affect the economic benefits measured by per capita GDP.

## 3. Institutional Background and Theoretical Analysis

### 3.1. Institutional Background

CETS can be traced back to the “*Kyoto Protocol*” in December 1997. The protocol proposed a market-based approach to greenhouse gases, known as CETS. It also advocated that all signatories should reduce emissions, but developed countries are greater than developing countries. Since then, developed countries, such as the UK, Germany, the EU, and Australia, have successively launched CETS, which means that CETS has changed from concept to practice. The EU CETS is the largest CETS in the world, who completed 80% of the global carbon trading volume. Furthermore, California and Tokyo have also established regional CETS. The existing CETS control local greenhouse gases and provide experience for the global CETS.

China needs to reduce emissions without harming the economy as a developing country. Therefore, the Chinese government has been committed to CETS in recent years. In October 2011, the Chinese government announced that CETS would pilot in seven regions, including Guangdong Province, Hubei Province, Beijing, Shanghai, Tianjin, Shenzhen, and Chongqing. From June 2013 to April 2014, the CETS was launched in seven pilot regions. Since then, the regional governments have continuously improved the supporting facilities of CETS, such as incentives and punishment system, cross-regional trading system, and offset system. At the same time, policies such as carbon mortgage, carbon finance, carbon funds, and carbon bonds have continuously strengthened the capital attributes of carbon emission rights. In December 2017, China began planning the national CETS. On July 16, 2021, the national CETS officially launched online trading. On the first day, the trading volume reached 4.1 million tons, with a turnover of 210.2 million yuan. So far, China's CETS has completed the spread from pilots to the entire nation. Although the national CETS only covers the power industry, it will radiate to electrolytic aluminum, cement, steel, petrochemical, chemical, papermaking, aviation, and other sectors in the future. At that time, the national CETS is expected to become the largest CETS in the world.

### 3.2. Theoretical Analysis

CETS is a market-based ER used to control nongreen behaviors of firms. New behaviorism theory holds that behavior depends not only on the perception of stimuli but also on behavior results. Similarly, whether firms can participate in CETS more actively depends on whether CETS has positive incentive effects. The Coase theorem emphasizes the property and market rules. The former makes public resources commodities, and the latter restrains participants' economic behavior [27]. CETS exerts incentive effects through the financial means of carbon emission trading. High-carbon firms can purchase carbon quotas to waive penalties for nonviolations. Low-carbon firms can sell carbon quotas to get additional economic benefits. In the end, a high level of emission reduction benefits can be achieved at a low economic cost. Moreover, Porter hypothesis argues that strict and flexible ER can promote economic growth while controlling pollution [10]. As an essential market-based regulatory tool, CETS uses market prices as a signal to enable firms to have higher flexibility [28]. Firms with more flexibility are more likely to increase productivity to alleviate and offset the additional costs of ER. Therefore, this article believes that CETS ultimately produces positive incentive effects. That is, CETS has significant emission reduction benefits and economic benefits.

**Figure 1:**
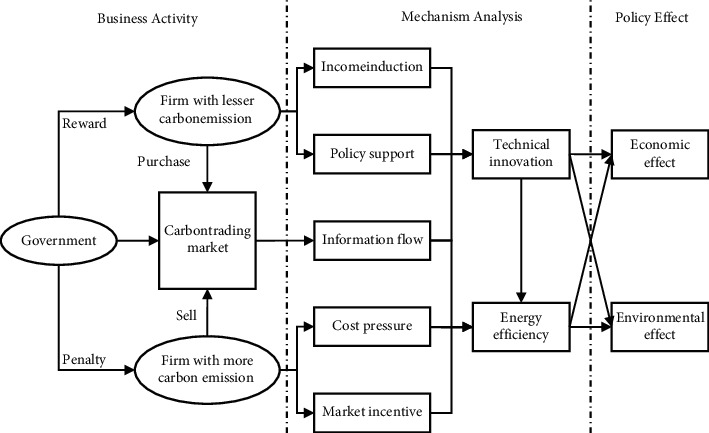
Theory frame.

According to behaviorism theory, to achieve the dual effects of economy and emission reduction, the behavior adopted by firms must be conducive to both economy and environment, namely, green behaviors. This article analyzes the path of CETS to achieve positive incentive effects from two common green behaviors, as shown in Figure 1.

1. CETS achieves economic and emission reduction benefits by promoting technological innovation. Because innovation has the characteristics of a long cycle, considerable investment, and high risk [29], whether a firm innovates depends on the external incentives it receives [21]. The analysis of CETS's policy benefits through technological innovation is as follows. (a) The surplus carbon quotas can be sold or used to offset the cost of violations. At this time, CETS provides firms with continuous and dynamic economic incentives. (b) CETS provides firms with market information about technological improvement, thereby reducing the uncertainty of technological innovation [30]. (c) CETS could increase firms' environmental and production costs as a legal pressure. As the pursuer of profit, firms' motivation to reduce cost by improving production technology will increase. Therefore, CETS can promote technological innovation and ultimately achieve economic and emission reduction benefits.
2. CETS achieves emission reduction and economic benefits by improving energy efficiency. China's pilot policy on CETS adopts means of total control. First, the total quotas of national carbon emissions are determined, and then, certain carbon quotas are allocated to specific firms. If firms emit more than their quotas, they need to buy quotas from the carbon market or face default penalties. (a) Carbon emissions mainly come from the combustion of energy. The most direct means of emission reduction is to reduce energy consumption [31]. Nevertheless, this crude means greatly damage the economy [32]. Improving energy efficiency means using less energy with the same output, reducing carbon emissions without sacrificing the economy. (b) CETS make carbon quotas rare commodities. It makes capital flow to energy-saving industries, conducive to improving energy utilization efficiency [33]. Therefore, CETS is conducive to energy efficiency and ultimately to reduce CO_2_ emissions and improve TFP.

## 4. Variables and Models

### 4.1. Variables

(1) CETS. China's pilot policy on CETS is an independent variable, represented by the dummy variable *TIME* × *TREAT. TIME* is a time dummy variable bounded by 2013 when the pilot policy started. The value is 0 from 2008 to 2012 and 1 from 2013 to 2017. *Treat* is a grouped dummy variable assigned to 1 if the province belongs to the pilot area and 0 otherwise. Among the seven pilot areas of CETS (Guangdong, Hubei, Beijing, Shanghai, Tianjin, Chongqing, and Shenzhen), Shenzhen is a city in Guangdong Province with a different administrative level from the other pilot areas, and its various data are a part of the Guangdong data. In addition, this article takes 30 provinces as samples for research. Therefore, the pilot areas mentioned in the subsequent study are Guangdong, Hubei, Beijing, Shanghai, Tianjin, and Chongqing.
(2) Emission reduction benefits. The emission reduction benefits measured by CO_2_ emissions is a dependent variable, and the data come from the author's calculation. CO_2_ mainly comes from the combustion of fossil energy. Therefore, the calculation of CO_2_ emissions usually uses energy consumption data, as shown in formula (1). Carbon emission factor provided by IPCC (as shown in Table 1).
  (1) $$\begin{array}{c} {\text{CO}}_{2}=\sum_{j=1}^{7}E_{j}\times {\text{NCV}}_{j}\times {\text{CEF}}_{j}, \end{array}$$
where, CO_2_ represents CO_2_ emissions, and *j* represents seven kinds of energy: coal, coke, gasoline, kerosene, diesel, fuel oil, and natural gas. *E* is energy consumption, *NCV* represents net calorific value, *CEF* represents carbon emission factor.
(3) Economic benefits. Economic efficiency measured by TFP is another dependent variable. DEA-Malmquist calculates this index based on provincial data. (a) Capital input. “Permanent inventory” is used to estimate capital stock. Firstly, according to Zhang et al. [34], the total fixed capital formation is selected as the investment indicator for the current year. Then, the actual investment with the constant price in 2000 is calculated through the investment product price index. Finally, the capital stock in 2008 is calculated according to Wang and Yan [35]. It can be referred to Wang et al. [36] to select the depreciation rate of each province. (b) Labor input. The number of employees is adopted as the labor input. (c) Energy input. Energy consumption is used to measure energy input. (d) Output. The real GDP with 2000 as the base period is selected as output.
(4) Control variables. Referring to Timothy et al. [2] and Akhmat et al. [31], the following variables are selected as control variables: the level of the service industry, optimization of industrial structure, level of foreign capital utilization, level of opening up, and level of education. The specific content is shown in Table 2.

### 4.2. Econometric Model

In order to test the emission reduction and economic effects of CETS, this paper constructs the DID model shown in formula (2).

(2) $$\begin{array}{c} Y_{\text{it}}=\alpha_{0}+\alpha_{1}\left({\text{TIME}}_{t}\times {\text{TREAT}}_{i}\right)+\beta X+\gamma_{t}+\mu_{i}+\varepsilon_{\text{it}}, \end{array}$$

where, *i* is the province, and *t* is the year. *Y* represents dependent variables, i.e., CO_2_ emissions and TFP. *TIME* × *TREAT* is the dummy variable representing China's pilot policy on CETS. The coefficient *α*_1_ is the emission reduction and economic benefits of CETS concerned in this article. *X* is the vector of control variables. *γ* is the time fixed effects, which controls the common time factors of all samples, such as business cycle, monetary policy, macroeconomic shock, and fiscal policy. *μ* is the individual fixed effects that control the characteristics of each sample that do not change with time, such as geographical characteristics, climate, and resource endowment. *ε* is the random error term.

## 5. Results and Discussions

### 5.1. Parallel Trend Analysis

**Figure 2:**
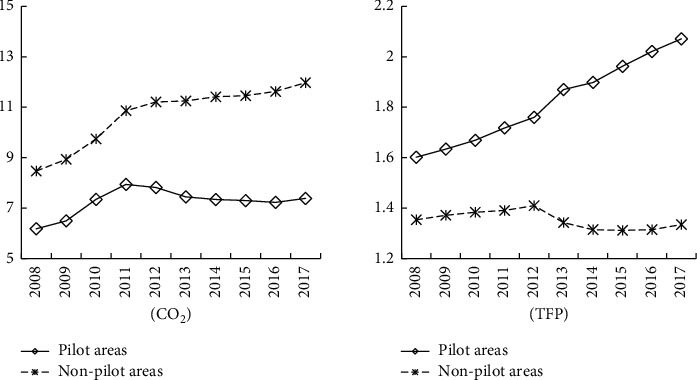
Means of CO_2_ emissions and TFP in pilot and nonpilot areas.

The trends diagram of CO_2_ emissions and TFP in pilot and nonpilot provinces are plotted in Figure 2. The emission reduction and economic benefits of pilot policy on CETS can be elementarily judged from the diagram. (a) CO_2_ emissions in nonpilot provinces are higher than that in pilot provinces. In 2008–2012, CO_2_ emissions in pilot and nonpilot areas showed an upward trend. After 2012, CO_2_ emissions in nonpilot areas changed slightly but remained on the rise, while CO_2_ emissions in pilot provinces showed a clear downward trend. (b) TFP in pilot provinces is higher than that in nonpilot areas. Before 2013, TPF in pilot and nonpilot provinces showed an upward trend. After 2013, the TFP growth of pilot provinces has increased, while nonpilot areas have shown a downward trend. Therefore, this article preliminarily speculated that compared with nonpilot areas, the decrease of CO_2_ emissions, and the increase of TFP in pilot provinces might be caused by the pilot policy on CETS in 2013.

### 5.2. Results of Benchmark Regression

The results of CETS's emission reduction and economic benefits are shown in Table 3. The coefficients of CETS's emission reduction and economic benefits are −0.189 and 0.346, respectively, significant at 1%. After adding control variables, columns (3) and (4) are the results. The regression results change slightly, but the sign and significance remain the same, which shows that the regression results are stable. The above results indicate that China's pilot policy on CETS has produced significant emission reduction and economic benefits. This study refutes the view of foreign scholars such as Allen et al. [37] that all marketization mechanisms in China are invalid. It is conducive to dispel the skepticism about China's marketization reform. As the central government's environmental and economic reform, CETS has political advantages in government guidance and market leadership. CETS provides an important direction for China to achieve green development through market-oriented means, under the dual pressure of environmental and economic.

### 5.3. Analysis of Dynamic Effects

The results of the benchmark regression only reflect the average emission reduction and average economic benefits of the pilot policy. In order to achieve the dynamic effects in CETS's emission reduction and economic benefits, a measurement model with reference to Jacobson et al. [38] is shown in formula (3), where *φ* represents a series of estimates for 2013–2017. *TIME*_*2013*_ is assigned as 1 in 2013 and 0 in other years. Similarly, *TIME*_*2014*_, *TIME*_*2015*_, *TIME*_*2016*_, and *TIME*_*2017*_ take 1 in 2014, 2015, 2016, and 2017, respectively, and 0 in other years. Other variables are defined following formula (2).

(3) $$\begin{array}{c} Y_{\text{it}}=\varphi_{0}+\sum_{t=2013}^{2017}\varphi_{t}\left({\text{TIME}}_{t}\times {\text{TREAT}}_{i}\right)+\phi X+\gamma_{t}+\mu_{i}+\varepsilon_{\text{it}}. \end{array}$$

Table 4 reports the results of dynamic analysis. It can be found that the absolute value and significance of *TIME* *×* *TREAT* gradually increase after the implementation of the pilot policy. It shows that the emission reduction and economic benefits of China's pilot policy on CETS increase with time. The possible reasons are as follows. CETS converts firms' emission reduction achievements into economic benefits through the carbon market. The longer the policy is implemented, the more complete the market construction will be, and the more emission reduction and economic benefits will be produced.

### 5.4. Results of Instrumental Variable

DID can subtly overcome the endogeneity by comparing treat and control groups, but this requires that the pilot areas be chosen randomly. However, this is not the case. The pilot work is not easy because the pilot policy is of great significance, and there are specific requirements for carbon trading technology and supporting measures. It is not completely random when the government determines pilot areas. That is to say, the estimation results of the DID may be disturbed by potential factors. Therefore, drawing on Hering and Poncet [39], the instrumental variable is used to overcome the endogeneity as much as possible.

Instrumental variables need to be related to endogenous variables and not associated with the stochastic disturbance team. CETS aims to stabilize temperature by reducing carbon emissions. Temperature is affected by region and climate and is an exogenous factor. Therefore, referring to the practice of Hu and Ding [40], the annual average temperature is taken as an instrumental variable of the pilot policy on CETS. Temperature data comes from each province's statistical yearbook and China meteorological yearbook. The two-stage least square is used for regression.

After adding the instrumental variable, the results are shown in Table 5, where *IV* represents the instrumental variables. In the regression results of the first stage, the coefficient of *TIME* *×* *IV*, the cross product of the instrumental variable and the time grouping variable, is significantly positive. It shows that the higher the temperature, the stricter the CETS is. The results of the weak instrumental variable show that the F value is 24.110, much higher than 10, rejecting the hypothesis of weak instrumental variables. In the regression results of the second stage, *TIME* *×* *TREAT*, the pilot policy on CETS, still has significant inhibition effects on CO_2_ emissions and significant promotion effects on TFP. The results indicate that the pilot policy on CETS still shows significant emission reduction and economic benefits after eliminating endogenous problems. That is, the results of the DID model are not caused by the bias in sample selection.

### 5.5. Results of Robustness Tests

**Figure 3:**
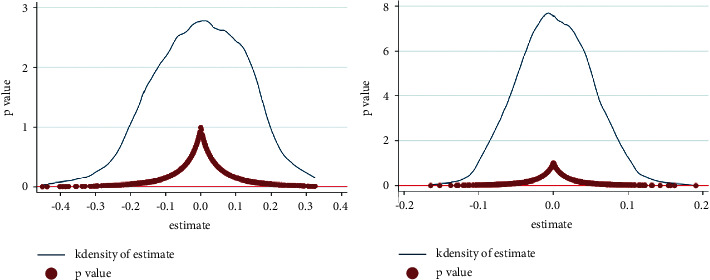
Results of placebo test.

1. To replace the dependent variable, CO_2_ emissions and TFP are replaced by pollutant emissions (*PE*) and GDP per capita (*PGDP*), respectively. Pollutant emissions are represented by the normalized indexes of total wastewater, exhaust gas, and general industrial solid waste production. Formula (2) is used for regression, and the results are shown in Table 6. The coefficient of pollutant emissions is still significantly negative, and the coefficient of economic benefit is still significantly positive. It suggests that the emission reduction and economic benefits of CETS do not depend on measures of dependent variables.
2. To change the regression method, tobit method is adopted to conduct regression analysis on formula (2) again, and the results are shown in Table 7. The pilot policy on CETS has a significantly negative impact on CO_2_ emissions and a significantly positive impact on TFP, consistent with the research results above. It shows that the regression method will not affect the estimation results. This result supports the robustness of the econometric model.
3. Dynamic window test. The dynamic effects of CTES's emission reduction and economic benefits have been analyzed above. However, it only focused on changes after policy implementation and could not compare the differences before and after implementation. Therefore, dynamic window tests are carried out based on Shi and Li [41]. In addition to dynamically analyzing benefits gaps, this test can also test whether the DID model is affected by time horizons. Specifically, with 2013 as the time node of the policy introduction. One year, two years, three years, and four years are selected as the time window width to re-regress formula (2). The test results are shown in Table 8. The effects direction of CETS on CO_2_ emissions and TFP does not change with the change of time window width. As the width of the time window increases, the significance of the coefficients of emission reduction and economic benefits keeps improving. It is consistent with the results of dynamic effects analysis.
4. Counterfactual test. The premise of DID is that the treatment and control groups are comparable. A year before the implementation of CTES is taken as the assumed impact point. If the assumed impact point coefficient is significant, there are significant differences between the experimental and control groups before implementing CETS. That is, the empirical model constructed in this article is not robust. On the contrary, if the coefficient is not significant, there is no significant difference between the experimental and control groups before implementing CETS. The difference between the two in the benchmark regression is caused by implementing CETS. The DID model constructed in this article is robust. In order to test this premise, referring to Hung et al. [42], the policy starting time of 2009, 2010, and 2011 is assumed as respectively, and formula (2) is used for regression. The results are shown in Table 9. The coefficient of *TIME* *×* *TREAT* is not significant when the start time is advanced to 2009, 2010, and 2011, respectively. It indicates that before the base year, the pilot policy of CETS cannot produce emission reduction and economic benefits. In other words, the actual policy year can indeed significantly reduce CO_2_ emissions and improve TFP. Therefore, the previous conclusion has strong robustness.
5. Placebo test. To further exclude the influence of unknown factors on the selection of pilot provinces and to ensure that the conclusions in this study are caused by the pilot policy on CETS, a placebo test is performed by randomly assigning pilot provinces [43]. In this article, 6 provinces are randomly selected as pilot areas of CETS from 30 provinces, and the other provinces are nonpilot areas. The placebo test should ensure that *TIME* *×* *TREAT*, the independent variable, has no impact on CO_2_ emissions and TFP. In other words, any significant findings will show that the results of this article are biased. 1000 random samples are taken using formula (2). The distribution of 1000 coefficients, and their *P* values are plotted in Figure 3. It can be seen that the distribution is mostly concentrated near the zero point, and the *P* value of most coefficients is bigger than 0.1. Therefore, the conclusions obtained in this article can pass the placebo test. The emission reduction and economic benefits of the pilot policy on CETS have no relationship with other unknown factors.
6. To exclude the impact of other policies. In 2007, the Chinese government launched a pilot policy on emissions trading systems in 11 provinces, including Tianjin, Hebei, Shanxi, Inner Mongolia, Jiangsu, Zhejiang, Henan, Hubei, Hunan, Chongqing, and Shaanxi. Studies have shown that the policy also contributes to the reduction of pollutant emissions and the improvement of TFP. In order to identify the effects of CETS accurately, it is necessary to exclude the interference of similar policies. In addition, reward and punishment policies related to energy-saving technologies are also important factors affecting economic and environmental benefits. China's Ministry of Finance stated in document No.7 in 2016 that the central finance would continue to allocate funds to reward new energy technology-related issues from 2016 to 2020. Therefore, formula (2) is used for re-regression after removing policy cross-region and cross-time samples. The results in Table 10 show that the coefficients of the two interaction terms are significant at the 1% level, and the influence direction of the CETS on these two dependent variables does not change. It indicates that the results are still robust after excluding the interference of other policies.

## 6. Further Analysis

### 6.1. Analysis of Mechanism

The above results show that China's pilot policy on CETS has positive incentive benefits. However, what behaviors should firms take under the stimulation of CETS to get positive incentive results? In order to verify the role of technological innovation and energy efficiency, the mediation model as shown in formulas (4) and (5) is constructed. The significance of the interaction terms' coefficient in formula (4) and the mediating variables' coefficient in formula (5) are the focus, where, *MEDIATOR* represents the mediating variable, namely, energy efficiency (*EE*) and technological innovation (*TI*). Other variables are defined as formula (2).

(4) $$\begin{array}{c} {\text{MEDIATOR}}_{it}=\theta_{0}+\theta_{1}\left({\text{TIME}}_{t}\times {\text{TREAT}}_{i}\right)+\lambda X+\gamma_{t}+\mu_{i}+\varepsilon_{it}, \end{array}$$

(5) $$\begin{array}{c} Y_{it}=\delta_{0}+\delta_{1}\left({\text{TIME}}_{t}\times {\text{TREAT}}_{i}\right)+\delta_{2}\;{\text{MEDIATOR}}_{it}+\eta X+\gamma_{t}+\mu_{i}+\varepsilon_{it}. \end{array}$$

The three-step method is used to test whether the mediating effects are significant. Firstly, the impact of CETS on CO_2_ emissions is significantly negative, and the impact on TFP is significantly positive (Table 3). Secondly, the effects of CETS on the mediating variable are tested. The results are significantly positive, as shown in columns (1) and (4) of Table 11. Finally, the effects of mediating variables on CO_2_ emissions and TFP are tested, respectively. As shown in Table 11, technological innovation and energy efficiency have a significantly negative impact on CO_2_ emissions and a significantly positive impact on TFP. Therefore, it can be concluded that CETS can stimulate firms to improve technological innovation and energy efficiency to obtain positive incentive effects.

### 6.2. Analysis of Heterogeneity

1. Resource endowment. According to the resource curse hypothesis, regions with abundant resources have greater cost advantages. Therefore, they are not sensitive to the compliance cost pressure and the economic incentives of CETS. Conversely, firms in poor resource endowment areas face higher illegal costs. They are more likely to gain additional economic benefits through carbon trading to cover environmental costs. A comprehensive index of the stock of 16 mineral resources, such as oil, natural gas, and coal, is used to measure the resource endowments of each province. The sample is divided according to the resource stock in 2012, one year before implementing the pilot policy on CETS. The provinces with resource stock higher than the average are defined as resource-rich provinces. The provinces with resource stock lower than or equal to the average are defined as resource-deficient provinces. In order to test the heterogeneity of the incentive effects of CETS under different resource endowments, formula (2) is used to regress the two samples separately. As shown in Table 12, CETS's emission reduction and economic benefits are greater in resource-deficient provinces than in resource-rich provinces. It suggests that the policy effects of CETS are also affected by the resource curse.
2. Environmental law enforcement. The implementation of CETS needs a solid legal system, such as collecting carbon emission information and the punishment of noncompliance with trading rules. In order to protect local interests, some local officials allow polluters to discharge carbon emissions illegally, which leads to the failure of carbon emission trading. Tu and Chen [44] argued that strict environmental enforcement is necessary for China's market-oriented ER to achieve Porter's benefits. In theory, the greater the intensity of environmental law enforcement is, the higher the illegal cost is. There will be fewer violations, such as leakage and nonperformance. Also, firms are more likely to follow CETS. The sample was divided by the number of environmental administrative penalty cases in each province in 2012 (the year before the policy was implemented). Provinces with lower than the mean are defined as lax provinces, while those with higher or equal to the mean are defined as strict provinces. In order to test the heterogeneity of the incentive benefits of CETS under different environmental law enforcement, formula (2) is used to regress the two samples separately. The results in Table 13 show that the positive incentive effects of CETS are greater in provinces with strict environmental law enforcement. It indicates that the effective enforcement of China's CETS needs the support of local governments, especially environmental law enforcement departments.

## 7. Conclusion

Taking China's pilot policy on CETS in 2013 as a natural experiment, a DID model was constructed to control the potential endogenous problems. Under the guidance of behavioral theory, a comprehensive study was conducted on the emission reduction benefits and economic benefits of China's CETS. The conclusions are as follows. (a) China's pilot CETS has produced significant emission reduction benefits and economic benefits. The results persisted after a series of robustness tests, including instrumental variable test, dynamic window test, counterfactual test, and placebo test of random sampling. (b) China's CETS can reduce CO_2_ emissions and improve TFP through technological innovation and energy efficiency. (c) The results of heterogeneity analysis show that resource-deficient provinces and provinces with strict environmental law enforcement are more sensitive to CETS and have greater emission reduction and economic benefits.

Future research could focus on the following aspects. (a) Future research can investigate the impact of CETS on energy efficiency, industrial structure, and firm strategy to explore the firm's other responding behaviors to CETS. (b) Consumers are part of greenhouse gas emitters, and the following researchers can include them when formulating a more comprehensive carbon reduction policy system.

## Figures and Tables

**Table 1:** Carbon emission factors of IPCC.

Energy types	Coal	Coke	Gasoline	Kerosene	Diesel	Fuel oil	Natural gas
NCV (kj/kg)	20908	28435	43070	43070	42652	43070	38931
CEF (kg/tj)	95333	107000	70000	71500	74100	77400	56100

**Table 2:** Variable design and description.

Variable types	Variable name	Code	Measure index
Independent variable	CETS	TIME × TREAT	China's pilot policy on CETS since 2013

Dependent variable	Emission reduction benefits	CO_2_	CO_2_ emissions
	Economic benefits	TFP	Total factor productivity

Control variable	Level of service industry	SI	Value-added service industry/GDP
	Optimization of industrial structure	OIS	Value-added tertiary industry/value-added secondary industry
	Level of foreign capital utilization	FDI	Actual utilization of FDI/GDP
	Level of opening up	OPEN	Total export/GDP
	Level of education	EDU	Total population of high school and above/total population at year end

Mediator variable	Technology innovation	TI	Turnover in the technology market
	Energy efficiency	EE	Total energy consumption/GDP

Note: the original data came from China Statistical Yearbook and China Environmental Statistical Yearbook. The data of TFP and EE were calculated by the authors.

**Table 3:** Results of benchmark regression.

VAR	(1)	(2)	(3)	(4)
	CO_2_	TFP	CO_2_	TFP
TIME × TREAT	−0.189^*∗∗∗*^	0.346^*∗∗∗*^	−0.151^*∗∗∗*^	0.213^*∗∗∗*^
	(0.028)	(0.051)	(0.028)	(0.050)
OIS			−0.003^*∗∗∗*^	0.003^*∗∗∗*^
			(0.001)	(0.001)
SI			0.009^*∗∗∗*^	−0.020^*∗∗∗*^
			(0.004)	(0.006)
FDI			−0.045^*∗∗∗*^	0.057^*∗∗*^
			(0.015)	(0.026)
OPEN			−0.003^*∗∗*^	−0.016^*∗∗∗*^
			(0.002)	(0.003)
EDU			0.050^*∗∗∗*^	−0.063^*∗∗∗*^
			(0.013)	(0.023)
CONS	7.790^*∗∗∗*^	2.164^*∗∗∗*^	8.063^*∗∗∗*^	2.945^*∗∗∗*^
	(0.037)	(0.066)	(0.184)	(0.325)
PROV	YES	YES	YES	YES
YEAR	YES	YES	YES	YES
*N*	300	300	300	300
*R* ^2^	0.985	0.783	0.988	0.826

**Table 4:** Results of dynamic effects.

VAR	(1)	(2)	(3)	(4)
	CO_2_	TFP	CO_2_	TFP
TIME_2013_ × TREAT	−0.137^*∗∗∗*^	0.233^*∗∗∗*^	−0.104^*∗∗*^	0.187^*∗∗*^
	(0.049)	(0.088)	(0.045)	(0.081)
TIME_2014_ × TREAT	−0.164^*∗∗∗*^	0.289^*∗∗∗*^	−0.141^*∗∗∗*^	0.233^*∗∗∗*^
	(0.049)	(0.088)	(0.045)	(0.081)
TIME_2015_ × TREAT	−0.176^*∗∗∗*^	0.356^*∗∗∗*^	−0.130^*∗∗∗*^	0.207^*∗∗*^
	(0.049)	(0.088)	(0.047)	(0.083)
TIME_2016_ × TREAT	−0.223^*∗∗∗*^	0.412^*∗∗∗*^	−0.192^*∗∗∗*^	0.215^*∗∗*^
	(0.049)	(0.088)	(0.048)	(0.085)
TIME_2017_ × TREAT	−0.243^*∗∗∗*^	0.441^*∗∗∗*^	−0.214^*∗∗∗*^	0.227^*∗∗∗*^
	(0.049)	(0.088)	(0.048)	(0.086)
OIS			−0.003^*∗∗∗*^	0.003^*∗∗∗*^
			(0.001)	(0.001)
SI			0.008^*∗∗*^	−0.020^*∗∗∗*^
			(0.004)	(0.006)
FDI			−0.046^*∗∗∗*^	0.057^*∗∗*^
			(0.015)	(0.026)
OPEN			−0.004^*∗∗*^	−0.015^*∗∗∗*^
			(0.002)	(0.003)
EDU			0.050^*∗∗∗*^	−0.064^*∗∗∗*^
			(0.013)	(0.023)
CONS	7.790^*∗∗∗*^	2.164^*∗∗∗*^	8.110^*∗∗∗*^	2.942^*∗∗∗*^
	(0.037)	(0.066)	(0.186)	(0.331)
PROV	YES	YES	YES	YES
YEAR	YES	YES	YES	YES
*N*	300	300	300	300
*R* ^2^	0.986	0.787	0.988	0.826

**Table 5:** Results of the instrumental variable.

VAR	First stage		Second stage	
	(1)	(2)	(3)	(4)
	TIME × TREAT	TIME × TREAT	CO_2_	TFP
TIME × IV	0.028^*∗∗∗*^	0.028^*∗∗∗*^		
	(0.006)	(0.006)		
TIME × TREAT			−0.210^*∗∗*^	0.525^*∗∗∗*^
			(0.089)	(0.167)
OIS	0.005^*∗∗∗*^	0.005^*∗∗∗*^	−0.002^*∗∗∗*^	0.002
	(0.001)	(0.001)	(0.001)	(0.001)
SI	−0.014^*∗*^	−0.014^*∗*^	0.008^*∗∗*^	−0.015^*∗∗*^
	(0.007)	(0.007)	(0.004)	(0.007)
FDI	−0.027	−0.027	−0.045^*∗∗∗*^	0.054^*∗∗*^
	(0.032)	(0.032)	(0.014)	(0.026)
OPEN	−0.010^*∗∗∗*^	−0.010^*∗∗∗*^	−0.004^*∗∗*^	−0.012^*∗∗∗*^
	(0.003)	(0.003)	(0.002)	(0.003)
EDU	−0.050^*∗*^	−0.050^*∗*^	0.046^*∗∗∗*^	−0.039
	(0.028)	(0.028)	(0.014)	(0.026)
CONS	0.002	0.002	8.088^*∗∗∗*^	2.816^*∗∗∗*^
	(0.401)	(0.401)	(0.174)	(0.329)
PROV	YES	YES	YES	YES
YEAR	YES	YES	YES	YES
*N*	300	300	300	300
*R* ^2^	0.652	0.652	0.988	0.799

**Table 6:** Results of replacing dependent variables.

VAR	(1)	(2)	(3)	(4)
	PE	PGDP	PE	PGDP
TIME × TREAT	−0.305^*∗∗∗*^	1.027^*∗∗∗*^	−0.178^*∗∗*^	0.754^*∗∗∗*^
	(0.071)	(0.134)	(0.070)	(0.122)
OIS			−0.006^*∗∗∗*^	−0.003
			(0.002)	(0.003)
SI			0.023^*∗∗*^	−0.034^*∗∗*^
			(0.009)	(0.015)
FDI			−0.175^*∗∗∗*^	0.053
			(0.036)	(0.064)
OPEN			0.003	−0.064^*∗∗∗*^
			(0.004)	(0.007)
EDU			0.086^*∗∗∗*^	−0.035
			(0.033)	(0.057)
CONS	−1.236^*∗∗∗*^	7.017^*∗∗∗*^	−0.823^*∗*^	11.598^*∗∗∗*^
	(0.093)	(0.175)	(0.461)	(0.803)
PROV	YES	YES	YES	YES
YEAR	YES	YES	YES	YES
*N*	300	300	300	300
*R* ^2^	0.949	0.967	0.957	0.977

**Table 7:** Results of changing econometric model.

VAR	(1)	(2)	(3)	(4)
	CO_2_	TFP	CO_2_	TFP
TIME × TREAT	−0.189^*∗∗∗*^	0.346^*∗∗∗*^	−0.151^*∗∗∗*^	0.213^*∗∗∗*^
	(0.026)	(0.047)	(0.026)	(0.046)
OIS			−0.003^*∗∗∗*^	0.003^*∗∗∗*^
			(0.001)	(0.001)
SI			0.009^*∗∗∗*^	−0.020^*∗∗∗*^
			(0.003)	(0.006)
FDI			−0.045^*∗∗∗*^	0.057^*∗∗*^
			(0.013)	(0.024)
OPEN			−0.003^*∗∗*^	−0.016^*∗∗∗*^
			(0.001)	(0.003)
EDU			0.050^*∗∗∗*^	−0.063^*∗∗∗*^
			(0.012)	(0.021)
CONS	7.790^*∗∗∗*^	2.164^*∗∗∗*^	8.063^*∗∗∗*^	2.945^*∗∗∗*^
	(0.034)	(0.062)	(0.169)	(0.300)
PROV	YES	YES	YES	YES
YEAR	YES	YES	YES	YES
*N*	300	300	300	300

**Table 8:** Results of dynamic window tests.

VAR	One year		Two years		Three years		Four years	
	(1)	(2)	(3)	(4)	(5)	(6)	(7)	(8)
	CO_2_	TFP	CO_2_	TFP	CO_2_	TFP	CO_2_	TFP
TIME × TREAT	−0.087^*∗∗∗*^	0.107^*∗∗*^	−0.117^*∗∗∗*^	0.153^*∗∗∗*^	−0.137^*∗∗∗*^	0.170^*∗∗∗*^	−0.150^*∗∗∗*^	0.198^*∗∗∗*^
	(0.028)	(0.047)	(0.027)	(0.048)	(0.027)	(0.051)	(0.028)	(0.052)
OIS	−0.001	0.005	−0.000	0.003^*∗*^	−0.002^*∗∗*^	0.003^*∗∗*^	−0.002^*∗∗∗*^	0.002^*∗∗*^
	(0.002)	(0.003)	(0.001)	(0.002)	(0.001)	(0.001)	(0.001)	(0.001)
SI	0.007	−0.033^*∗∗*^	0.004	−0.031^*∗∗∗*^	0.006	−0.025^*∗∗∗*^	0.006	−0.017^*∗∗*^
	(0.010)	(0.016)	(0.005)	(0.009)	(0.004)	(0.008)	(0.004)	(0.007)
FDI	−0.005	0.122^*∗∗*^	−0.015	0.131^*∗∗∗*^	−0.028^*∗*^	0.069^*∗∗*^	−0.040^*∗∗∗*^	0.066^*∗∗*^
	(0.033)	(0.055)	(0.027)	(0.049)	(0.017)	(0.031)	(0.015)	(0.027)
OPEN	−0.003	−0.012	−0.002	−0.012^*∗∗*^	−0.002	−0.015^*∗∗∗*^	−0.002	−0.017^*∗∗∗*^
	(0.005)	(0.008)	(0.003)	(0.005)	(0.002)	(0.004)	(0.002)	(0.003)
EDU	0.031	0.006	0.033^*∗∗*^	−0.020	0.037^*∗∗∗*^	−0.045^*∗*^	0.040^*∗∗∗*^	−0.055^*∗∗*^
	(0.020)	(0.033)	(0.016)	(0.028)	(0.013)	(0.024)	(0.014)	(0.025)
CONS	7.642^*∗∗∗*^	3.138^*∗∗∗*^	7.813^*∗∗∗*^	3.494^*∗∗∗*^	7.994^*∗∗∗*^	3.281^*∗∗∗*^	8.069^*∗∗∗*^	2.875^*∗∗∗*^
	(0.479)	(0.796)	(0.265)	(0.473)	(0.213)	(0.397)	(0.208)	(0.380)
PROV	YES	YES	YES	YES	YES	YES	YES	YES
YEAR	YES	YES	YES	YES	YES	YES	YES	YES
*N*	90	90	150	150	210	210	270	270
*R* ^2^	0.998	0.969	0.995	0.928	0.992	0.885	0.989	0.848

**Table 9:** Results of the counterfactual test.

VAR	2009		2010		2011	
	(1)	(2)	(3)	(4)	(5)	(6)
	CO_2_	TFP	CO_2_	TFP	CO_2_	TFP
TIME × TREAT	−0.046	0.092	−0.029	0.094	−0.038	0.100
	(0.032)	(0.033)	(0.026)	(0.067)	(0.026)	(0.067)
OIS	−0.008^*∗∗∗*^	0.005	−0.007^*∗∗∗*^	0.000	−0.007^*∗∗∗*^	−0.000
	(0.002)	(0.001)	(0.002)	(0.001)	(0.002)	(0.001)
SI	0.021^*∗∗∗*^	0.009	0.020^*∗∗∗*^	0.009^*∗*^	0.020^*∗∗∗*^	0.010^*∗*^
	(0.006)	(0.005)	(0.006)	(0.005)	(0.006)	(0.005)
FDI	−0.076^*∗∗∗*^	0.025^*∗*^	−0.078^*∗∗∗*^	0.026^*∗*^	−0.078^*∗∗∗*^	0.026^*∗*^
	(0.024)	(0.014)	(0.024)	(0.014)	(0.024)	(0.014)
OPEN	−0.005^*∗∗*^	0.004^*∗∗∗*^	−0.005^*∗∗*^	0.004^*∗∗∗*^	−0.005^*∗∗*^	0.004^*∗∗∗*^
	(0.002)	(0.001)	(0.002)	(0.001)	(0.002)	(0.001)
EDU	0.038^*∗∗∗*^	0.005	0.037^*∗∗∗*^	0.005	0.033^*∗∗*^	0.009
	(0.014)	(0.020)	(0.014)	(0.020)	(0.014)	(0.020)
CONS	8.966^*∗∗∗*^	0.986^*∗∗∗*^	8.924^*∗∗∗*^	0.971^*∗∗∗*^	8.892^*∗∗∗*^	0.957^*∗∗∗*^
	(0.276)	(0.134)	(0.277)	(0.129)	(0.278)	(0.128)
PROV	YES	YES	YES	YES	YES	YES
YEAR	YES	YES	YES	YES	YES	YES
*N*	150	150	150	150	150	150
*R* ^2^	0.995	0.940	0.995	0.943	0.995	0.943

**Table 10:** Results after excluding other policies.

VAR	(1)	(2)	(3)	(4)
	CO_2_	TFP	CO_2_	TFP
TIME × TREAT	−0.196^*∗∗∗*^	0.389^*∗∗∗*^	−0.134^*∗∗∗*^	0.187^*∗∗∗*^
	(0.040)	(0.059)	(0.044)	(0.064)
OIS			−0.003^*∗∗∗*^	0.005^*∗∗∗*^
			(0.001)	(0.001)
SI			0.010^*∗∗*^	−0.031^*∗∗∗*^
			(0.005)	(0.007)
FDI			−0.068^*∗∗∗*^	0.033
			(0.020)	(0.030)
OPEN			−0.003	−0.013^*∗∗∗*^
			(0.002)	(0.003)
EDU			0.056^*∗∗∗*^	−0.043^*∗∗*^
			(0.014)	(0.020)
CONS	7.865^*∗∗∗*^	2.031^*∗∗∗*^	8.156^*∗∗∗*^	2.971^*∗∗∗*^
	(0.038)	(0.056)	(0.236)	(0.346)
PROV	YES	YES	YES	YES
YEAR	YES	YES	YES	YES
*N*	216	216	216	216
*R* ^2^	0.989	0.828	0.991	0.860

**Table 11:** Results of mechanism test.

	(1)	(2)	(3)	(4)	(5)	(6)
	TI	CO_2_	TFP	EE	CO_2_	TFP
TIME × TREAT	4.018^*∗∗∗*^	−0.090^*∗∗∗*^	0.103^*∗*^	0.331^*∗∗∗*^	−0.022^*∗*^	0.062^*∗*^
	(0.528)	(0.030)	(0.053)	(0.039)	(0.027)	(0.048)
EE					−0.390^*∗∗∗*^	0.704^*∗∗∗*^
					(0.038)	(0.066)
TI		−0.015^*∗∗∗*^	0.027^*∗∗∗*^			
		(0.003)	(0.006)			
OIS	0.095^*∗∗∗*^	−0.001^*∗∗*^	0.000	0.002^*∗∗∗*^	−0.002^*∗∗∗*^	0.001
	(0.011)	(0.001)	(0.001)	(0.001)	(0.001)	(0.001)
SI	−0.506^*∗∗∗*^	0.002	−0.006	−0.031^*∗∗∗*^	−0.003	0.002
	(0.066)	(0.004)	(0.007)	(0.005)	(0.003)	(0.006)
FDI	0.538^*∗*^	−0.037^*∗∗∗*^	0.042^*∗*^	0.090^*∗∗∗*^	−0.010	−0.006
	(0.274)	(0.014)	(0.025)	(0.020)	(0.013)	(0.022)
OPEN	−0.004	−0.003^*∗∗*^	−0.015^*∗∗∗*^	−0.004^*∗*^	−0.005^*∗∗∗*^	−0.013^*∗∗∗*^
	(0.029)	(0.001)	(0.003)	(0.002)	(0.001)	(0.002)
EDU	0.598^*∗∗*^	0.059^*∗∗∗*^	−0.079^*∗∗∗*^	−0.049^*∗∗∗*^	0.031^*∗∗∗*^	−0.029
	(0.246)	(0.013)	(0.022)	(0.018)	(0.011)	(0.020)
CONS	25.549^*∗∗∗*^	8.454^*∗∗∗*^	2.248^*∗∗∗*^	3.611^*∗∗∗*^	9.472^*∗∗∗*^	0.403
	(3.461)	(0.194)	(0.343)	(0.256)	(0.206)	(0.361)
PROV	YES	YES	YES	YES	YES	YES
YEAR	YES	YES	YES	YES	YES	YES
*N*	300	300	300	300	300	300
*R* ^2^	0.912	0.989	0.840	0.964	0.991	0.879

**Table 12:** Results of heterogeneity in resource endowment.

VAR	Resource-deficient provinces		Resource-rich provinces	
	(1)	(2)	(3)	(4)
	CO_2_	TFP	CO_2_	TFP
TIME × TREAT	−0.140^*∗∗∗*^	0.271^*∗∗∗*^	−0.195^*∗∗∗*^	−0.078
	(0.031)	(0.064)	(0.063)	(0.062)
OIS	−0.002^*∗∗∗*^	0.002^*∗*^	−0.006^*∗∗*^	−0.006^*∗∗*^
	(0.001)	(0.001)	(0.003)	(0.003)
SI	0.008^*∗*^	−0.027^*∗∗∗*^	0.020^*∗*^	0.024^*∗∗*^
	(0.004)	(0.009)	(0.012)	(0.011)
FDI	−0.052^*∗∗∗*^	0.101^*∗∗∗*^	−0.041	−0.021
	(0.018)	(0.037)	(0.025)	(0.025)
OPEN	−0.002	−0.016^*∗∗∗*^	−0.014^*∗∗∗*^	0.006
	(0.002)	(0.003)	(0.005)	(0.005)
EDU	0.007	−0.041	0.071^*∗∗∗*^	−0.036^*∗*^
	(0.018)	(0.037)	(0.022)	(0.021)
CONS	8.062^*∗∗∗*^	3.556^*∗∗∗*^	8.817^*∗∗∗*^	1.135^*∗∗∗*^
	(0.255)	(0.520)	(0.228)	(0.222)
PROV	YES	YES	YES	YES
YEAR	YES	YES	YES	YES
*N*	180	180	120	120
*R* ^2^	0.992	0.841	0.975	0.773

**Table 13:** Results of heterogeneity in environmental enforcement.

VAR	Lax provinces		Strict provinces	
	(1)	(2)	(3)	(4)
	CO_2_	TFP	CO_2_	TFP
TIME × TREAT	−0.026	0.037	−0.150^*∗∗∗*^	0.127^*∗∗*^
	(0.037)	(0.087)	(0.036)	(0.060)
OIS	−0.005^*∗∗∗*^	0.008^*∗∗∗*^	−0.000	−0.001
	(0.001)	(0.001)	(0.001)	(0.002)
SI	0.038^*∗∗∗*^	−0.083^*∗∗∗*^	−0.001	0.003
	(0.006)	(0.014)	(0.005)	(0.008)
FDI	0.043	−0.148^*∗∗*^	−0.047^*∗∗∗*^	0.065^*∗∗*^
	(0.026)	(0.062)	(0.016)	(0.027)
OPEN	−0.003	−0.021^*∗∗∗*^	−0.003^*∗*^	−0.017^*∗∗∗*^
	(0.002)	(0.005)	(0.002)	(0.003)
EDU	0.001	0.035	0.068^*∗∗∗*^	−0.136^*∗∗∗*^
	(0.016)	(0.037)	(0.017)	(0.028)
CONS	6.722^*∗∗∗*^	6.100^*∗∗∗*^	9.013^*∗∗∗*^	1.627^*∗∗∗*^
	(0.338)	(0.797)	(0.125)	(0.209)
PROV	YES	YES	YES	YES
YEAR	YES	YES	YES	YES
*N*	220	220	80	80
*R* ^2^	0.995	0.913	0.985	0.786

## Data Availability

The data of this paper can be accessed by contacting the authors.
